# Reporting of Methodologic Information on Trial Registries for Quality Assessment: A Study of Trial Records Retrieved from the WHO Search Portal

**DOI:** 10.1371/journal.pone.0012484

**Published:** 2010-08-31

**Authors:** Ludovic Reveiz, An-Wen Chan, Karmela Krleža-Jerić, Carlos Eduardo Granados, Mariona Pinart, Itziar Etxeandia, Diego Rada, Monserrat Martinez, Xavier Bonfill, Andrés Felipe Cardona

**Affiliations:** 1 Research Institute, Sanitas University Foundation, Bogotá, Colombia; 2 Women's College Research Institute, University of Toronto, Women's College Hospital, Toronto, Canada; 3 Knowledge Synthesis and Exchange Branch, Knowledge Translation Portfolio, Canadian Institutes of Health Research, Ottawa, Canada; 4 Research Institute, National University of Colombia, Bogotá, Colombia; 5 Experimental Studies, Airway Disease Section, National Heart and Lung Institute, Imperial College London, London, United Kingdom; 6 Clinical Epidemiology Unit, Cruces Hospital & Osteba-Basque Office for HTA, Department of Health, Bilbao, Spain; 7 Unidad de Investigación de Atención Primaria, Bilbao, Spain; 8 Institut de Recerca Biomèdica de Lleida - Universitat de Lleida, Catalonia, Spain; 9 Servicio de Epidemiología Clínica y Salud Pública, Hospital de la Santa Creu i Sant Pau, Barcelona, Spain; 10 Clinical and Translational Oncology Group, Fundación Santa Fe de Bogotá, Bogotá, Colombia; Swiss Paraplegic Research, Switzerland

## Abstract

**Background:**

Although randomized clinical trials (RCTs) are considered the gold standard of evidence, their reporting is often suboptimal. Trial registries have the potential to contribute important methodologic information for critical appraisal of study results.

**Methods and Findings:**

The objective of the study was to evaluate the reporting of key methodologic study characteristics in trial registries. We identified a random sample (n = 265) of actively recruiting RCTs using the World Health Organization International Clinical Trials Registry Platform (ICTRP) search portal in 2008. We assessed the reporting of relevant domains from the Cochrane Collaboration's ‘Risk of bias’ tool and other key methodological aspects. Our primary outcomes were the proportion of registry records with adequate reporting of random sequence generation, allocation concealment, blinding, and trial outcomes. Two reviewers independently assessed each record. Weighted overall proportions in the ICTRP search portal for adequate reporting of sequence generation, allocation concealment, blinding (including and excluding open label RCT) and primary outcomes were 5.7% (95% CI 3.0–8.4%), 1.4% (0–2.8%), 41% (35–47%), 8.4% (4.1–13%), and 66% (60–72%), respectively. The proportion of adequately reported RCTs was higher for registries that used specific methodological fields for describing methods of randomization and allocation concealment compared to registries that did not. Concerning other key methodological aspects, weighted overall proportions of RCTs with adequately reported items were as follows: eligibility criteria (81%), secondary outcomes (46%), harm (5%) follow-up duration (62%), description of the interventions (53%) and sample size calculation (1%).

**Conclusions:**

Trial registries currently contain limited methodologic information about registered RCTs. In order to permit adequate critical appraisal of trial results reported in journals and registries, trial registries should consider requesting details on key RCT methods to complement journal publications. Full protocols remain the most comprehensive source of methodologic information and should be made publicly available.

## Introduction

Critical appraisal of randomized clinical trials (RCTs) relies on the availability of adequate information about study design and conduct. Based on methods described in published journal articles, RCTs are often considered to be “inadequately reported” or to have “high or unclear risk of bias” depending on the instruments used to evaluate them [1]. Therefore, even if an RCT has been well-designed and conducted, a lack of adequate reporting in the publication may decrease its perceived quality and strength of evidence for guiding clinical practice [1], [2].

Full protocols are a particularly valuable source of information about the design and conduct of RCTs [1], [3]. However, given that protocols are often not publicly available, trial registries currently constitute the main public source of basic protocol information.

The number of registries and registered clinical trials has been increasing since 2004, after the requirement for public registration at study inception was introduced by the International Committee of Medical Journal Editors (ICMJE) [4]–[7]. There is general agreement about the minimum protocol information that should be registered for a trial, as defined by the 20-item World Health Organization (WHO) Registration Data Set [8]. However, the limited methodologic information contained in the WHO data set does not permit full appraisal of trial quality. The ICMJE statement [4] expressed that even if acceptable completion of data fields was an important concern, many entries in a publicly accessible registries did not provide meaningful information in some key data fields. The World Health Organization International Clinical Trials Registry Platform (ICTRP) considers that the registration of ongoing trials is useful among others because it “may lead to improvements in the quality of clinical trials by making it possible to identify potential problems (such as problematic randomization methods) early in the research process” (http://www.who.int/ictrp/trial_reg/en/index.html).

Although some registries have included additional items to improve the reporting of the trials, those items are not compulsory. Consequently, there is a gap between the WHO 20-items and the information needed to adequately appraise a trial. For example, the WHO data set contains a specific item for the “study type” which should include the type of study (interventional or observational) and details on the study design (method of allocation, masking, methods of randomization and the phase (if applicable) [8]. However it is unclear how much detail should be reported on methods of randomization, allocation concealment and blinding. This is understandable because trial registries were initially conceived to identify the existence of a trial and not to provide all the required information concerning methodological issues.

However, in addition to the overall benefits of increased transparency and identification of suppressed trial results, registries have the potential to provide valuable methodologic information that is necessary for critical appraisal of trial results. Given the recent policies mandating public disclosure of results for relevant registered trials [9], [10], information on trial methods will be increasingly important to reliably interpret the trial results.

We assessed the quality of methodological information available for ongoing RCTs registered in six WHO Primary Registries and ClinicalTrials.gov in 2008.

## Methods

We used the WHO/ICRTP (ICTRP) Search Portal to identify a random sample of RCTs registered from January 1 to December 12, 2008 and open for recruitment on December 12, 2008. At the time of our study, the ICTRP Search Portal provided access to data from ClinicalTrials.gov and six WHO primary registries (Australian New Zealand Clinical Trials Registry (ANZCTR), the Chinese Clinical Trial Register, the Clinical Trials Registry - India, the German Clinical Trials Register, International Standard Randomised Controlled Trial Number Register (ISRCTN), and the Netherlands National Trial Register). Each record was screened by one reviewer (LR) to identify RCTs. The record was included if it explicitly used the word ‘random’ or variations thereof to describe the allocation method.

We extracted data on key methodologic items from each registry record using an evidence based source to define domains developed by the Cochrane Collaboration [1]. The tool consists of six domains (sequence generation, allocation concealment, blinding, incomplete outcome data, selective outcome reporting and ‘other issues’) for assessing the quality of an RCT based primarily on published reports. The description of each domain provides a general risk of bias in the included randomized trials as well as any important flaws in the studies [1]. Although some domains can only be evaluated once the trial is published, others are relevant at the registration stage and were designated as the primary outcomes of our study: the proportions of RCT records with adequate reporting of the methods of random sequence generation, allocation concealment, blinding and outcomes (primary, secondary, and harms outcomes). While harms can be primary or secondary outcomes, we decided to collect specific information on harms because they are frequently underreported. We also evaluated descriptions of the follow-up period, trial interventions and sample size calculations. For each methodological item, we defined adequate reporting based on the Cochrane Collaboration Risk of Bias tool [1], the Consolidated Standards of Reporting Trials (CONSORT) [11], the WHO Trial Registration Data Set (http://www.who.int/ictrp/network/trds/en/index.html) and the instructions for registrants provided by registries (Table 1). An item was classified as inadequately reported if the reporting was unclear (i.e. some useful information provided, but insufficient detail to meet the definition of adequacy) or absent (i.e. no useful information provided) (Table 1). Finally, we also collected information on the type of intervention (drugs, procedures, behavior/education, devices, vaccines, and combined) and the type of funding (industry versus non-industry).

**Table 1:** Criteria for defining adequate reporting.

Item	Criteria
Sequence generation	Description of the process used to generate the random allocation sequence, such as:1. Referring to a random number table; 2. Using a computer random number generator; Coin tossing; Shuffling cards or envelopes; Throwing dice; 3. Drawing of lots; Minimization.
Allocation concealment	Description of the method used to conceal the allocation sequence from participants and investigators such that it could not be predicted in advance (e.g., Central allocation (including telephone, web-based and pharmacy-controlled randomization); sequentially numbered drug containers of identical appearance; sequentially numbered, opaque, sealed envelopes).
Blinding	1. Explicit statement that the study is open label; or 2. For blinded studies, all of the following: a) A description of who was masked: the individuals receiving the treatment/s;the individuals administering the treatment/s; the individuals assessing the outcomes; the individuals analysing the results/data b) Complete description ensuring that blinding could not be broken (e.g., “double blind, double dummy”; “tablets or capsules are indistinguishable in all aspects of their outward appearance”)
Primary and secondary outcomes	Specific variable, metric, and measurement timepoints of interest for all primary outcomes (e.g., “% with Beck depression score >10 at 6 months” rather than just “depression”).
Harms outcomes	Description of outcomes related to adverse events and abnormalities of laboratory tests (laboratory-determined toxicity), as well as the procedure to collect the information: Specific variables and timepoints of collection for harms were provided Instrument(s) to be used for the assessment/ measurement, where possible.
Follow-up duration	Explicit statement of the length of follow-up. When the outcome is time to an event, the follow-up duration is variable for each participant and may not be specifically known.
Interventions	Specific names of the interventions assigned to trial participants, and a description of other relevant intervention details as applicable (e.g., dose, duration, mode of administration, etc)
Sample size calculation	Description of key elements of the sample size calculation: The outcome variable used; The alpha (Type I) error level and the statistical power (or the beta [Type II] error level); The clinically important difference between the intervention groups; For binary outcomes, the estimated results in each group; For continuous outcomes, the variance, standard deviation or standard error of the measurements.
Number of participants in each arm	The number of participants in each arm.
Inclusion and exclusion criteria	Explicit definition of eligibility criteria, including age and sex.

The total number of records for all study designs is shown in Table 2. The ICTRP search portal does not differentiate records of RCTs from other study designs. We therefore estimated that 50% of these trial records were RCTs based on a random pilot sample of 100 records, as well an advanced search strategy using the word ‘random’ or variations thereof in the title or intervention field of all records from the ICTRP search portal. We sought to ensure adequate representation across registries by including all available RCTs from those registries with few records (the Chinese, Indian and German registries). For each of the other registries, we estimated the proportion of RCTs with adequate reporting of random sequence generation or allocation concealment to be less than 12% based on a pilot sample of 100 records. We then calculated the sample size required for each of these larger registries to yield an estimated prevalence of adequate reporting with 95% confidence, 80% power and 7% precision (Table 2). Each record in the sample was screened to ensure that it was an RCT (the word ‘random’ or variations thereof). For RCTs that were registered on multiple registries, we randomly selected only one record for inclusion.

**Table 2:** Identification of study sample based on total number of recruiting studies and estimated number of RCTs registered from January 1 to December 12, 2008.

Trial registry	Number of recruiting studies	Weight of estimated number of RCTs in the ICTRP search portal	Number of recruiting RCTs included in our study sample
ANZCTR	226	2.4%	49
Chinese Clinical Trials register	71	0.1%	6
Clinical Trial Registry-India	45	0.5%	21
Clinicaltrials.gov	8503	89.6%	81
German Clinical Trials Register	10	0.1%	5
ISRCTN	541	5.7%	63
Netherlands NTR	153	1.6%	40
Total	9549	100%	265

Two independent reviewers extracted information from each trial, with discrepancies resolved by discussion with a third evaluator. Five of the seven data extractors participated in an advanced method of systematic review course organized by the Ibero-American Cochrane Centre in Madrid and therefore received the same training on the Cochrane Collaboration Risk of Bias tool and other issues of RCT assessment. The two evaluators had experience in the used the Cochrane tool. Data were analyzed descriptively as weighted and raw proportions using SPSS 15.0, and Chi-square tests were used to determine associations between categorical variables.

## Results

From the 7 clinical trial registries, we included a total of 265 RCT records with principal registrants from 35 countries. In terms of blinding, 141 RCTs were reported as blinded (53%), 101 as open label (38%) and 23 (9%) records contained insufficient information for judgment; four trials had one open label arm and one blinded arm. The types of interventions included drugs (60%), procedures (15%), behavior modification/education/counseling (13%), devices (4.2%), and combination of interventions -e.g. drug vs. procedure- (4%), vaccines (1.1%), and other (2.3%).

Overall, the proportion of adequate reporting varied by methodologic item from the Cochrane Collaboration Risk of Bias tool: random sequence generation (weighted proportion 5.7%, 95% CI 3.0–8.4%), allocation concealment (1.4%, 0–2.8%), blinding (41%, 35–47% including open label RCTs; 8.4%, 4.1–13% excluding open label RCTs), primary outcomes (66%, 60–72%), secondary outcomes (46%,40–52%) and harms outcomes (5%, 2–8%) (Table S1). Weighted proportions were calculated using data from Table 2. Most records reported no useful information for allocation concealment (97.9%) and harm (89.5%), and had insufficient detail for blinding (86.2%, excluding open label RCTs) and primary outcome measures (32%). One record stated that the trial was blinded in the Methods section and open in the Summary section. Examples of adequate and unclear reporting are shown in Table 3.

**Table 3:** Examples of adequate and unclear reporting.

Item	Adequate reporting	Unclear reporting
Sequence generation:	*“Randomization table using computer software”*	“*Randomization*”
Allocation concealment:	“Central randomization by computer”; “Sequentially numbered, sealed, opaque envelopes”	“Sealed envelopes”; “Envelopes”
Blinding:	“Blinded/ masking used”; and who is blinded: “The people receiving the treatment”… “administering the treatments” “assessing the outcomes” “analyzing the results”…”placebo oral tablets designed to look, smell and taste similar than…”,	“Blind”; “Single blind”
Interventions:	*“Finasteride 5 mg PO once daily for 8 weeks…”*	“Levofloxacin” vs. “gentamicin”; “Treatment as usual” vs. “Behavioral: case management”; “Stress” vs. “no-stress”
Outcomes:	“Progression-free survival which is measured by regular CT (computerised tomography) scans prior to treatment, every six weeks during chemotherapy, and every two months after chemotherapy until the lung cancer has progressedTimepoint: After 460 progression events have occurred over all of the participants in the study (after 460 patients have shown progression of their lung cancer)”	“Morbidity of chemotherapy and surgery”; “The primary and secondary outcome measures will be measured after the completion of the trial.” (no further details); “Improvement in metabolic profile and histology at 6 months”
Eligibility criteria	---	“Patients with symptomatic atrial fibrillation..” and “other inclusion criteria”

Concerning other methodological items, weighted overall proportions of trial records with adequately reported items were variable (Table S2). Adequate reporting of eligibility criteria, follow up duration and study interventions were 81%, 62% and 53% respectively. Reporting of details of sample size calculations was particularly poor, with adequate descriptions in only 1% of records. Although the target sample size was reported for 97% of trials, the number of participants in each study arm was stated in only 7%. Only four records provided a link to the full study protocol.

Reporting of methodologic items varied substantially across registries, with two registries (Australian New Zealand Clinical Trials Registry and the Clinical Trials Registry – India) having higher proportions of trials with adequate reporting of most items than the others (Table S1).

Characteristics of the data entry fields also varied across clinical trial registries (Tables S1 and S2). The proportion of specific fields for all 11 methodologic items ranged from 37% (4/11) to 82% (9/11) within each registry. ANZCTR, Clinical Trials Registry – India, and the Chinese Clinical Trial Register were the only registries that offered specific fields for random sequence generation and allocation concealment. Few registries provided fields for reporting sample size calculations (Clinical Trials Registry – India) or the planned number of participants in each study arm (Chinese Clinical Trial Register).

27% of RCTs from the sample were funded by industry. Clinicaltrials.gov contained a significantly higher proportion of industry funded RCTs compared to the WHO Primary Registries (43% vs. 20%; p<0.001). In an exploratory analysis, no significant differences were found for adequate reporting of random sequence generation, allocation concealment, blinding and primary outcomes between industry and non-industry RCTs. We found no significant difference in adequate reporting of study interventions when comparing trials of drug interventions versus all other intervention types.

## Discussion

### Main findings

A key obstacle in the assessment of trial quality is the lack of available information about study design and conduct [1], [12]. Despite important initiatives to improve reporting, such as CONSORT [10], methodologic descriptions in trial publications often provide inadequate detail and do not necessarily reflect the way the trial was conducted [2]. Trial registries offer an added source of protocol information to complement journal publications and to track the existence of an RCT. There is general agreement about the minimum protocol information that should be registered for a trial, as defined by the 20-item WHO Registration Data Set [8] supported by ICMJE, other medical journals [13] and the Ottawa group [12]. However, our findings reveal that the amount of methodologic information available for critical appraisal of a trial is low overall and varies between registries. Our results are consistent with previous literature demonstrating that the WHO Registration Data Set items are often incompletely registered by trialists and do not encompass key aspects of trial design and planned conduct [14]–[18]. Nonetheless, when adequately reported, the information available in registries can be useful to evaluate the risk of bias and other key methodological aspects related to internal and external validity.

There are various possible explanations for the low prevalence of adequate reporting. A number of the assessed items (i.e. allocation concealment, method of randomization, sample size calculations) are not explicitly part of the WHO 20-item Trial Registration Data Set (TRDS) or ICMJE requirements [4], [8]. Although they are not mandatory items for the registries included in the study, they are essential for assessing the quality and the risk of bias of RCTs [1], [11]. To encourage registration in these early years, most registries have focused on ensuring adherence to the 20 WHO TDRS items. However, our findings are helpful in highlighting the lack of information for other essential items or a more detailed description of the TRDS items.

Clearly if there is no field for a given item on the registry, then it cannot be recorded. This was most relevant for reporting of sample size calculations and the number of participants in each study arm. The reporting of key methodological information is also influenced in part by the way registries asks registrants to enter their data. For example, the ANZCTR, the Indian and Chinese Clinical Trials Registries –have specific fields for describing the methods of randomization and allocation concealment whereas other registries have general (“Methods” Fields) or restrictive coded fields (restricted to randomized, non-randomized, observational etc). Most registries offer specific coded fields for blinding (open label, single blind or double blind) or a description of masked roles (subject, caregiver, investigator, outcomes assessor). However, a specific free text field or a more detailed coded field is not available to allow a more detailed description of how blinding was achieved.

In a cross-section of trials registered at ClinicalTrials.gov, Ross et al reported that nearly 100% of ClinicalTrials.gov records provided mandatory data elements (title, sponsor, condition studied, design, type, phase, and intervention and population studied), although the quality of information was not assessed. Conversely, reporting of optional data elements varied (66% for primary outcome measures; 56% for secondary outcome measures) [19].

There were important differences among those registries that incorporated key methodological fields and those that did not. We found that registries having specific fields for some items obtained more relevant information for critical appraisal than registries with general open and or coded fields. Registries should consider including specific data entry fields to record methodologic items that have been associated with bias [1]. It seems that specific fields for certain domains (e.g. “describe the allocation concealment procedure”) have more impact than open fields (e.g. “describe methods”). The usefulness of the information for the public, clinicians, systematic reviewers, and other stakeholders interested in trial results could be improved by including a few additional methodologic fields.

Another reason for poor reporting on registry records is that the information may not even available in the source document, the full protocol. Previous studies have shown that methodologic information is often inadequately described in RCT protocols [20]–[25]. In addition, if the individuals registering the trial have an administrative rather than a scientific background, they may not have sufficient methodologic knowledge to properly register the information.

To complement the limited methodologic information available on trial registries, the Ottawa Statement (http://ottawagroup.ohri.ca.) and others have recommended public disclosure of the full protocol to enable reliable interpretation of trial findings [3], [12], [26], [27]. However, few protocols are currently publicly available and their content is variable. To improve transparency, the SPIRIT initiative (Standard Protocol Items for Randomized Trials) has been developing evidence-based recommendations on the essential information to describe in protocols [28]. With the recent move towards mandatory registration and results disclosure on public databases [9], [10], [29] and ongoing discussion regarding international standards such as PROCTOR (Public Reporting of Clinical Trial Outcomes and Results) [30], improved public availability of methodologic information will become increasingly important to place results into their proper context.

### Study limitations

Due to technical limitations of the databases and duplicate registrations, it was not possible to determine exactly the total number of RCTs in the registries without manually reviewing all 9549 records registered during our study period. We thus had to estimate the total number of RCTs based on a pilot sample. It is unlikely that our calculation of weighted proportions would have been significantly affected by any inaccuracy, since the vast majority of records were from one registry (ClinicalTrials.gov).

Another limitation is that the assessment of some items was inherently subjective. For example, assessment of reporting of eligibility criteria was limited by data extractors not being intimately familiar with the clinical topic of every RCT. In some cases, it was also difficult to determine adequate reporting for non-drug interventions (i.e. surgery, education, counselling, devices). However, we used a low threshold to classify records as adequately reported for these uncertain circumstances. We also used duplicate data extraction to reduce bias, and involved a third individual when necessary.

### Conclusion

Reporting of methodologic information on trial registries has not been a focus of early registration requirements, and consequently the quality of reporting of trial methods in registry records is poor overall. It is imperative that disclosure of trial results in public databases or journal publications be accompanied by sufficient methodologic information to fully appraise them. Considering that widespread implementation of trial registration is relatively recent, registries can continue to learn from each other, from empiric studies, and from their own internal evaluations to improve the reporting of trial methods. Full trial protocols remain a key source of methodologic information, and should be made publicly available.

## Supporting Information

Table S1 Characteristics of trial registry fields and proportion of trial registry records with adequate reporting of information to evaluate four “risk of bias” tool domains.
(0.06 MB DOC)

Table S2 Characteristics of trial registry fields and proportion of trial registry records with adequate reporting of other key methodological items.
(0.05 MB DOC)
