# Supplementary material for: Reporting of Methodologic Information on Trial Registries for Quality Assessment: A Study of Trial Records Retrieved from the WHO Search Portal
Source: PLoS One. 2010 Aug 31;5(8):e12484. doi: 10.1371/journal.pone.0012484 (PMC2930852; doi:10.1371/journal.pone.0012484)
Supplement: Table S1 — Characteristics of trial registry fields and proportion of trial registry records with adequate reporting of information to evaluate four “risk of bias” tool domains. (0.06 MB DOC) [file pone.0012484.s001.doc]

Table S1. Characteristics of trial registry fields and proportion of trial registry records with adequate reporting of information to evaluate four “risk of bias” tool domains.

| **Item field /Registry** | **Australian New Zealand Clinical Trials Registry**  **(N=49)** | **Chinese Clinical Trial Register**  **(N=6)** | **Clinical Trials Registry of India**  **(N=21)** | **Clinicaltrials.gov**  **(N=81)** | **German Clinical Trials Register**  **(N=5)** | **ISRCTN**  **(N=63)** | **Netherlands National Trial Register**  **(N=40)** | **Overall weighted proportion (% and 95% CI)** |
| --- | --- | --- | --- | --- | --- | --- | --- | --- |
| **RANDOM SEQUENCE GENERATION** | | | | | | | |  |
| Specific field | Yes | Yes | Yes | No (under ‘Allocation’) | No (under ‘Allocation’) | No (under ‘Study design’) | No (under ‘Randomised’) |  |
| Description in instructions for registrants | Detailed | None | Detailed | None | Mentioned briefly | Mentioned briefly | Mentioned briefly |  |
| Type of data entry | Free text | Restricted choices | Restricted choices (‘Permuted block’, ‘fixed’) | Restricted choices (‘Randomized’, ‘non-randomized’, ‘N/A’) and optional free text | Restricted choices | Free text | Restricted choices |  |
| **Adequate reporting N(%)** | **38 (78%)** | **0 (0%)** | **18 (86%)** | **2 (3%)** | **0 (0%)** | **2 (3%)** | **1 (2.5%)** | **5.7% (3.0-8.4%)** |
| **ALLOCATION CONCEALMENT** | | | | | | | |  |
| Specific field | Yes | Yes | Yes | No | No | No | No |  |
| Instructions for registrants | Detailed | None | Detailed | None | None | Mentioned briefly | None |  |
| Type of data entry | Free text | Free text | Coded field | - | - | - | - |  |
| **Adequate reporting N(%)** | **27 (55%)** | **0 (0%)** | **13 (62%)** | **0 (0%)** | **0 (0%)** | **0 (0%)** | **0 (0%)** | **1.4% (0-2.8%)** |
| **BLINDING** | | | | | | | |  |
| Specific field | Yes | Yes | Yes | Yes | Yes | No | Yes |  |
| Instructions for registrants | Yes | No | Yes | Yes | Yes | Mentioned briefly | Yes |  |
| Type of data entry | a. Masking/ blinding: coded field  b. Who are masked: coded field  c. For a description to ensure that blinding could not be broken: no field | a. Masking/ blinding: coded field  b. Who are masked: coded field  c. For a description to ensure that blinding could not be broken: open and coded | a. Masking/ blinding: coded field  b. Who are masked: coded field  c. For a description to ensure that blinding could not be broken: no field | a. Masking/ blinding: coded field  b. Who are masked: coded field  c. For a description to ensure that blinding could not be broken: no  field | a. Masking/ blinding: coded field  b. Who are masked: Not a field  c. For a description to ensure that blinding could not be broken: no  field | Under “Study design”: open field limited to 200 characters | a. Masking/ blinding: coded field  b. Who are masked: coded field  c. For a description to ensure that blinding could not be broken: no field |  |
| **Adequate reporting**  **(including open label RCTs)**  **N(%)** | **35 (71%)** | **0 (0%)** | **14 (67%)** | **33 (41%)** | **2 (40%)** | **23 (37%)** | **19 (48%)** | **41% (35-47%)** |
| **Adequate reporting**  **(excluding open label RCTs)**  **N(%)** | **14/28 (50%)** | **0/6 (0%)** | **4/11 (36%)** | **4/52 (8%)** | **0/3 (0%)** | **2/42 (5%)** | **1/22 (5%)** | **8.4% (4.1-13%)** |
| **PRIMARY OUTCOMES** | | | | | | | |  |
| Specific field | Yes | Yes | Yes | Yes | Yes | Yes | Yes |  |
| Instructions for registrants | Yes | No | Yes | Yes | Yes | Yes | Yes |  |
| Type of data entry | Free text | Free text | Free text. Timepoint for each outcome: | Free text | Free text | Free text | Free text |  |
| **Adequate reporting N(%)** | 43 (88%) | 3 (50%) | 21(100%) | 53 (65%) | 2 (40%) | 42 (67%) | 26 (65%) | **66% (60-72%)** |
| **SECONDARY OUTCOMES** | | | | | | | |  |
| Specific field | Yes | Yes | Yes | Yes | Yes | Yes | Yes |  |
| Instructions for registrants | Yes | No | Yes | Yes | Yes | Yes | Yes |  |
| Type of data entry | Free text | Free text | Free text. Timepoint for each outcome: | Free text | Free text | Free text | Free text |  |
| **Adequate reporting N(%)** | **43 (88%)** | **3 (50%)** | **19 (90%)** | **34 (42%)** | **2 (40%)** | **40 (63%)** | **20 (50%)** | **46% (40-52%)** |
| **ADVERSE EVENTS** | | | | | | | |  |
| Specific field | No | No | No | A field if the outcome is related to safety issue. | No | No | No |  |
| Instructions for registrants | No | No | No | No | No | No | No |  |
| Type of data entry | - | - | - | Open text | - | - | - |  |
| **Adequate reporting N(%)** | **7 (14%)** | **0 (0%)** | **4 (19%)** | **4 (5%)** | **0 (0%)** | **4 (6%)** | **2 (5%)** | **5% (2-8%)** |
