# Supplementary material for: Reporting of Methodologic Information on Trial Registries for Quality Assessment: A Study of Trial Records Retrieved from the WHO Search Portal
Source: PLoS One. 2010 Aug 31;5(8):e12484. doi: 10.1371/journal.pone.0012484 (PMC2930852; doi:10.1371/journal.pone.0012484)
Supplement: Table S2 — Characteristics of trial registry fields and proportion of trial registry records with adequate reporting of other key methodological items. (0.05 MB DOC) [file pone.0012484.s002.doc]

Table S2. Characteristics of trial registry fields and proportion of trial registry records with adequate reporting of other key methodological items.

| **Item field /Registry** | **Australian New Zealand Clinical Trials Registry**  **(N=49)** | **Chinese Clinical Trial Register**  **(N=6)** | **Clinical Trials Registry of India**  **(N=21)** | **Clinicaltrials.gov**  **(N=81)** | **German Clinical Trials Register**  **(N=5)** | | **ISRCTN**  **(N=63)** | **Netherlands National Trial Register**  **(N=40)** | **Overall weighted proportion (% and 95% CI)** |
| --- | --- | --- | --- | --- | --- | --- | --- | --- | --- |
| **ELIGIBILITY CRITERIA** | | | | | | | | |  |
| Specific field | Yes | Yes | Yes | Yes | Yes | | Yes | Yes |  |
| Instructions for registrants | Yes | No | Yes | Yes | Yes | | Yes | Yes |  |
| Type of data entry | Free text | Free text | Free text | Free text | | Free text | Free text | Free text |  |
| **Adequate reporting N(%)** | **44 (90%)** | **4/ (67%)** | **21 (100%)** | **62 (77%)** | | **4 (80%)** | **55 (87%)** | **33**  **(83%)** | **81% (76-85%)** |
| **TIME TO FOLLOW UP** | | | | | | | | |  |
| Specific field | Yes | Yes | Yes | Yes | No | | No | No |  |
| Instructions for registrants | Yes | No | Mentioned briefly | Yes | No | | Mentioned briefly | No |  |
| Type of data entry | Open text | Open text | Open text | Open text | - | | - | - |  |
| **Adequate reporting N(%)** | **46 (94%)** | **0 (0%)** | **20 (95%)** | **48 (59%)** | **1 (20%)** | | **52 (83%)** | **19 (48%)** | **62% (56-68%)** |
| **INTERVENTIONS** | | | | | | | | |  |
| Specific field | Yes | Yes | Yes | Yes | Yes | | Yes | Yes |  |
| Instructions for registrants | Yes | No | Yes | Yes | Yes | | Yes | Yes |  |
| Type of data entry | Free text and coded fields | Free text | Free text | Free text | Free text | | Free text | Free text |  |
| **Adequate reporting N(%)** | **39 (80%)** | **2 (33%)** | **11 (52%)** | **41 (51%)** | **0 (0%)** | | **44 (70%)** | **15 (38%)** | **53% (47-59%)** |
| **SAMPLE SIZE CALCULATION** | | | | | | | | |  |
| Specific field | No | Yes | No | No | No | | No | No |  |
| Instructions for registrants | No | No | No | No | No | | No | No |  |
| Type of data entry | - | Free text | - | - | - | | - | - |  |
| **Adequate reporting N(%)** | **0 (0%)** | **0 (0%)** | **0 (0%)** | **1 (1%)** | **1 (20%)** | | **0 (0%)** | **0 (0%)** | **1% (0-2%)** |
| **NUMBER OF PARTICIPANTS IN EACH ARM** | | | | | | | | |  |
| Specific field | No. Only target sample size | No. Only target sample size | Yes | No. Only target sample size | No. Only target sample size | | No. Only target sample size | No. Only target sample size |  |
| Instructions for registrants | No | No | No | No | No | | Mentioned briefly | No |  |
| Type of data entry | - | - | Free text | - | - | | - | - |  |
| **Adequate reporting N(%)** | **22 (45%)** | **6 (100%)** | **18 (86%)** | **4 (5%)** | **0 (0%)** | | **8 (13%)** | **1 (3%)** | **7% (4-10%)** |
